# Neutrophils remain detrimentally active in hydroxyurea-treated patients with sickle cell disease

**DOI:** 10.1371/journal.pone.0226583

**Published:** 2019-12-23

**Authors:** Emilia Alina Barbu, Venina M. Dominical, Laurel Mendelsohn, Swee Lay Thein

**Affiliations:** 1 Sickle Cell Branch, National Heart, Lung, and Blood Institute, NIH, Bethesda, MD, United States of America; 2 Flow Cytometry Core Facility, National Heart, Lung, and Blood Institute, NIH, Bethesda, MD, United States of America; Hospital for Sick Children, CANADA

## Abstract

Neutrophilia is a feature of sickle cell disease (SCD) that has been consistently correlated with clinical severity and has been shown to remain highly activated even at steady state. In addition to induction of fetal hemoglobin (HbF), hydroxyurea (HU) leads to reduction in neutrophil count and their adhesion properties, which contributes to the clinical efficacy of HU in SCD. Although HU reduces the frequency and severity of acute vaso-occlusive crises (VOCs) and chest syndrome, HU therapy does not abolish these acute clinical events. In this study we investigated whether neutrophils in SCD patients whilst on HU therapy retained features of detrimental pro-inflammatory activity. Freshly isolated neutrophils from SCD patients on HU therapy at steady state and from ethnic-matched healthy controls were evaluated ex vivo for their degranulation response and production of neutrophil extracellular traps (NETs). Unstimulated SCD patient neutrophils already produced NETs within 30 minutes, compared to none for healthy neutrophils, and by 4 hours, these neutrophils produced significantly more NETs than the control neutrophils (P = 0.0079**). Higher numbers of neutrophils from SCD patients also showed higher degree of degranulation-related intracellular features compared to healthy neutrophils, including rough-textured cellular membranes (P = 0.03*), double-positivity for F-Actin and CD63 (P = 0.02*) and re-located CD63 within cytoplasm more efficiently than their healthy counterparts (P = 0.02*). The neutrophils from SCD donors released more myeloperoxidase (P = 0.02*) in the absence of any trigger. Our data showed that neutrophils from patients with SCD at steady state remained active during hydroxyurea treatment and are likely to be able to contribute to the SCD pro-inflammatory environment.

## Introduction

Sickle cell disease (SCD) is a heritable blood disorder caused by the presence of hemoglobin S (HbS) due to a point mutation in the β-hemoglobin (Hb) gene. The fundamental event that underlies the complex pathophysiology and multi-systemic consequences of SCD is the polymerization of HbS under low oxygen tension. Polymerization of the de-oxygenated-HbS alters the structure and function of the red blood cells (RBCs). These damaged (typically sickled shaped) RBCs are highly adhesive with a very shortened life-span and cause the chronic hemolysis and recurrent acute painful vaso-occlusive crises (VOC), hallmarks of SCD.[1] These events trigger a cascade of pro-inflammatory activity that also involve neutrophils, platelets and vascular endothelium. Neutrophils have a central role in VOCs through their interactions with both the erythrocytes and the endothelium; neutrophil count and their activity have been consistently correlated with disease severity.[2, 3]

Hydroxyurea has been used to treat SCD for >30 years and has been shown to reduce the frequency and severity of acute clinical events. While induction of HbF production is the major contributor to its clinical efficacy, HU therapy also reduces neutrophil count and neutrophil adherence to vascular endothelium, as demonstrated in both patients and sickle mouse models.[4–6]

Neutrophils’ efficient cytotoxic mechanisms can also be activated as part of a sterile inflammatory response that can be harmful to the host if not properly controlled. Formation of Neutrophil Extracellular Traps (NETs), a feature that enhances the anti-microbial effectiveness, has also been described in sterile pathologies with a major inflammatory component, such as autoimmune diseases, trauma or cancers.[7–9]

Although NETs presence might be relevant for SCD pathophysiology, they can also mediate significant vascular damage [10–12] and limited information is currently available on features of neutrophils that could have detrimental activity in SCD, such as NETs and NETosis, and dysregulated neutrophil degranulation. Neutrophil granules contain products (myeloperoxidase, elastase, lactoferrin, hydrolases, complement activators) that possess not only microbicidal but also have highly cytotoxic effects.[13, 14] Increased azurophilic granule activity has not yet been confirmed directly in neutrophils from patients with SCD although high plasma circulating levels of elastase and lactoferrin have been measured, suggesting increased product release from the neutrophil stores.[15]

In this study we showed that neutrophils from HU-treated SCD patients produced NETs and degranulate ex vivo spontaneously unlike healthy neutrophils. Functional responses of these neutrophils to high concentrations of hemin, however, were not significantly increased over those of the healthy neutrophils. The data suggest that, although reduced in numbers, neutrophils in SCD patients whilst on HU therapy, retain their overactivity neutrophils that can be detrimental to the inflammatory milieu of SCD.

## Materials and methods

### Study participants and definitions

Sickle cell disease patients with HbSS genotype (N = 17), HbSC genotype (N = 1) and HbS- β-thalassemia (N = 1) provided whole blood samples at steady state. The “steady state” was defined as the usual state of health of the patient, outside of an acute painful episode, and throughout this manuscript referred to as “SS” neutrophils. Patients were excluded when they were <18 or >80 years of age, were pregnant or had a history of blood transfusion 4 weeks prior to the blood donation. Written informed consent was obtained from both patients and ethnic-matched healthy controls through the study protocol NCT00047996 approved by The National Institutes of Health Institutional Review Board. The study complies with the Helsinki Declaration (1975, as revised 2008). 19 patients with SCD (15 on HU and 4 off HU treatment), and 11 ethnic-matched healthy volunteers were recruited for the study. Due to limitations of time and neutrophil quantity, not all isolated neutrophils could be used for all 3 types of studies that included NETosis visualization using imaging flow cytometry (IFC), NETosis as visualized by microscopy and early activation studies (CD63/Actin assays by IFC and ELISA assay for MPO). Only 4 of the 11 healthy samples (#2, #4, #5, and #6) (see Table A in S1 Table) and 2 of the 19 SCD samples (#1 and #8) (see Table B in S1 Table) were adequate for all 3 assays.

### Isolation and in vitro activation of neutrophils

Whole blood was collected at the same time in sodium heparin vacutainers from SCD subjects and healthy volunteers. After centrifugation on Ficoll-Paque Plus (GE Healthcare, IL), neutrophils were gently lifted from the top of RBC layer with a sterile 1-ml pipette and re-suspended in ice-cold DPBS; any contaminating RBCs were lysed by adding ice-cold water for 30 seconds and then restoring osmolarity with 0.6M KCl. Purified neutrophils were used for experiments within 2 hours of isolation. For the NETs experiments, neutrophils were incubated with RPMI or 20 μM hemin [16] for 7, 15, and 30 minutes (for imaging flow cytometry) or up to 4 hours (for fluorescence microscopy). For the activation experiments (IFC and ELISA) neutrophils were incubated for 30 minutes with RPMI or 20 μM hemin. Reactions were stopped with 4% paraformaldehyde (PFA) for 30 minutes at room temperature.

### Visualization using imaging flow cytometry (IFC)

IFC was used to visualize early nuclear and cellular changes of NETosis and neutrophil degranulation. To visualize nuclear and cellular changes, the PFA-fixed neutrophils were sequentially stained for CD66b, histone 4 citrulline 3 (H4cit3), MPO (myeloperoxidase) and DNA. ImageStream Mark II imaging flow cytometer (Millipore Sigma, Seattle, WA, USA) was used to acquire 20,000 events per test. Gating strategy and analysis features with IDEAS software are described in S1 Fig. Bright Detail Intensity_R3 (BDI_R3) feature identified normal nuclei as high intensity Hoechst positive spots with a radius of 3 pixels or less, and decondensed nuclei as wide, fuzzy areas of low intensity color. Neutrophils with damaged cell membranes were identified by plotting Standard Deviation feature on the side scatter (SSC channel) against Modulation in Brightfield as shown in S1 Fig. Lobe Count feature was used to identify changes in the number of nuclear lobes based on nuclear imaging and to quantify the number of neutrophils with unsegmented (1-lobe) nuclei. Neutrophils with super-condensed 1-lobe nuclei were identified by plotting nucleus/whole cell Area versus Morphology Area (Hoechst stain) as shown in S1 Fig. For the degranulation-related assays, the fixed neutrophils were next permeabilized and stained with anti-CD63-APC, ActinGreen488 and Hoechst.

### Visualization using fluorescence microscopy

All fluorescence microscopy experiments utilized a BZ-X710 All-in-One Fluorescence Microscope (Keyence, Osaka, Japan). NETs were visualized with elastase-Alexa Fluor 488, MPO-PE and DAPI nuclear staining; the staining protocol, modified from that described in Kahlenberg et al [17] is detailed in the supplemental material. At least 10 fields per treatment at 20x magnification (scale bar at 50 μm) were acquired in the Multi-color Image Capturing Mode. The number of overlaid DAPI and elastase positive strands (i.e. NETs) were counted in all acquired fields and shown as average number of NETs per field ± S.D.

### Myeloperoxidase assay

Freshly purified neutrophils were incubated with RPMI or 20 μM hemin in 96-well plates for 30 minutes at 37°C. The supernatant was collected, centrifuged twice to remove particles and then stored at -80°C. The supernatants were then assayed with a Human Myeloperoxidase Quantikine ELISA Kit from R&D Systems according to the manufacturer’s instruction.

### Statistics

Data is presented as mean±S.D., or dot plots±S.D. Statistical analyses were performed with PRISM7 software (Graph Pad Software, CA). Wilcoxon signed-rank test and Mann-Whitney tests were used for two group comparisons. *P<0.05, **P<0.01, ***P<0.001, ****P<0.0001.

## Results

### Neutrophils from subjects with SCD are primed towards NETs production

We quantified the ability of freshly purified neutrophils from patients with SCD to form NETs relative to that of healthy neutrophils, in the absence of a NETS-specific stimulus, and in the presence of hemin. Hemin is a stable form of heme which is elevated in hemolytic environment, including SCD. Free circulating heme has been associated with NETs formation in sickle mouse models and also in humans with SCD, the latter indirectly using sickle plasma as a stimulus on healthy neutrophils.[16]

**Fig 1:**
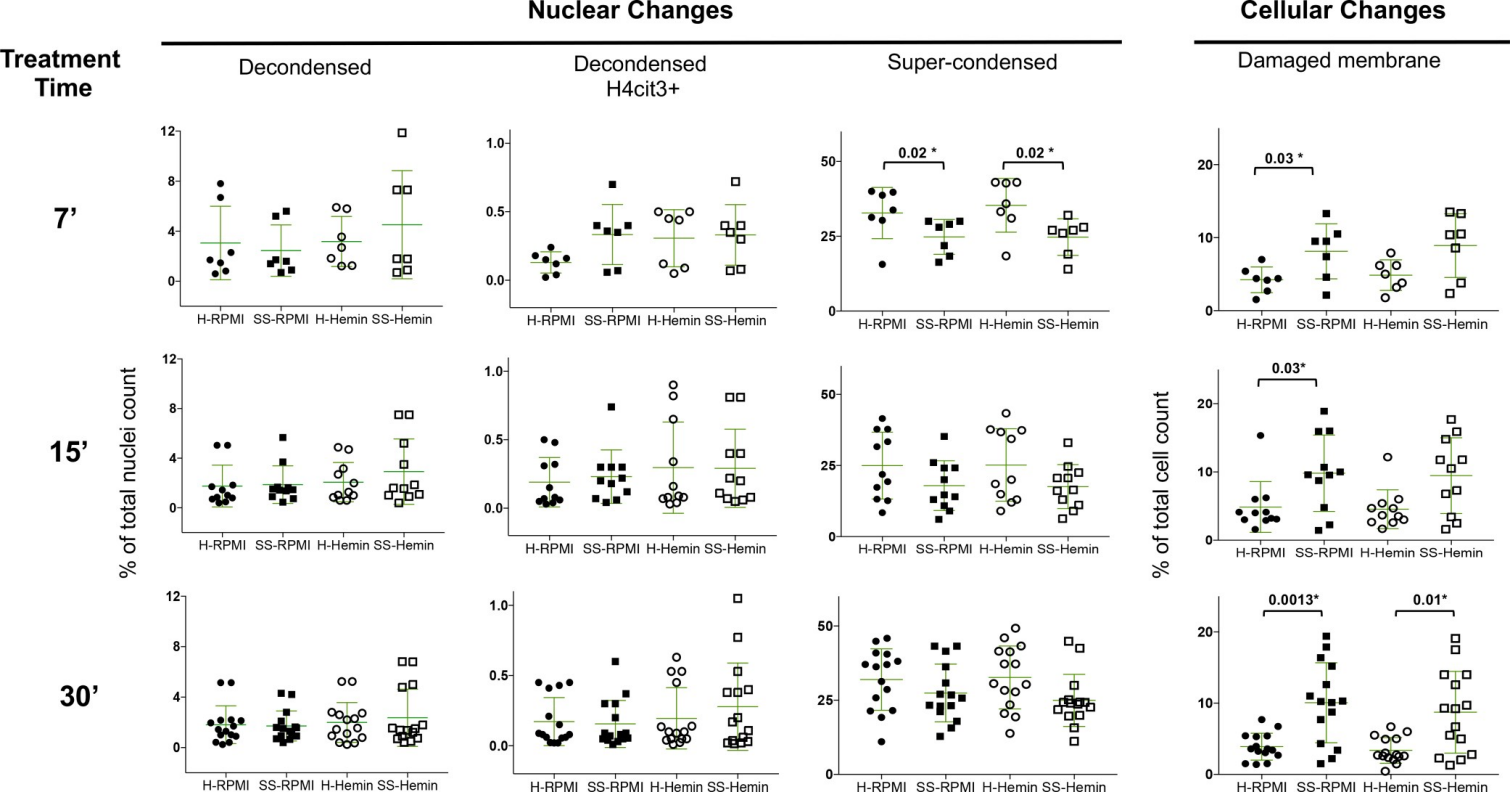
NETs-related nuclear and cellular changes occur in neutrophils from SCD patients in the absence of a NETs-specific stimulus and in the presence of hemin. Purified neutrophils from healthy donors (H) or SCD patients in steady-state (SS) were left untreated in RPMI or treated with 20 μM hemin for the specified times (7, 15 or 30 minutes). Stimulation was stopped with PFA and the fixed cells were stained for CD66b, H4cit3, MPO and DNA. Specific nuclear changes related with NETosis (nuclear decondensation in the presence or absence of histone H4 citrullination) or apoptosis (nuclear super-condensation), as well as cell membrane damage were assessed using imaging flow cytometry. Variable number of experimental repeats were conducted for each time point (7 minutes: N = 7: 15 minutes: N = 11; 30 minutes: N = 15). Data presented as dot plots±S.D., significance calculated with Mann-Whitney test. Note that SS refers to steady-state, and not the SCD genotype SS.

IFC was used to probe the morphology of untreated and hemin-treated neutrophils, prior to the release of the DNA-scaffolded strands. Neutrophils from 9 healthy volunteers and 12 SCD patients were tested. We evaluated cellular and nuclear changes after treatment periods ranging from 7 minutes to 30 minutes, to make sure any fast response from the SCD neutrophils was not missed. Within 7 minutes, the number of untreated SCD neutrophils with uneven and rough cell surfaces (possibly with damaged cell membranes) was significantly increased in comparison to the healthy neutrophils and remained so at 15 and 30 minutes (Fig 1 and S2 Table). Addition of hemin did not potentiate this feature in the SCD or in the healthy neutrophils at 7 or 15 minutes, but significantly increased it in the neutrophils from the SCD patients following 30 minutes of treatment (P = 0.01*). (Fig 1). At 7 minutes and in the absence of a stimulus, higher numbers of neutrophils from SCD patients showed nuclear decondensation in the presence of histone H4 citrullination compared to healthy neutrophils (0.33±0.21 vs 0.13±0.07), although the increase was not statistically significant. Overall nuclear decondensation was comparable between healthy and neutrophils from the SCD donors throughout the 30 minutes with or without hemin-mediated activation (Fig 1 and S2 Table). At 7 minutes, fewer SCD neutrophils had un-segmented and super-condensed nuclei compared to the healthy neutrophils (25±5.83 vs 33±8.55, P = 0.02*) but by 15 minutes the changes were similar in both SCD and healthy neutrophils, with or without addition of hemin (Fig 1). At these relatively short treatment time periods the addition of high concentration of hemin did not appear to enhance any of the nuclear or cellular features in neutrophils from SCD patients above that observed in healthy neutrophils.

### Neutrophils from SCD patients show increased ex vivo NETs production

**Fig 2:**
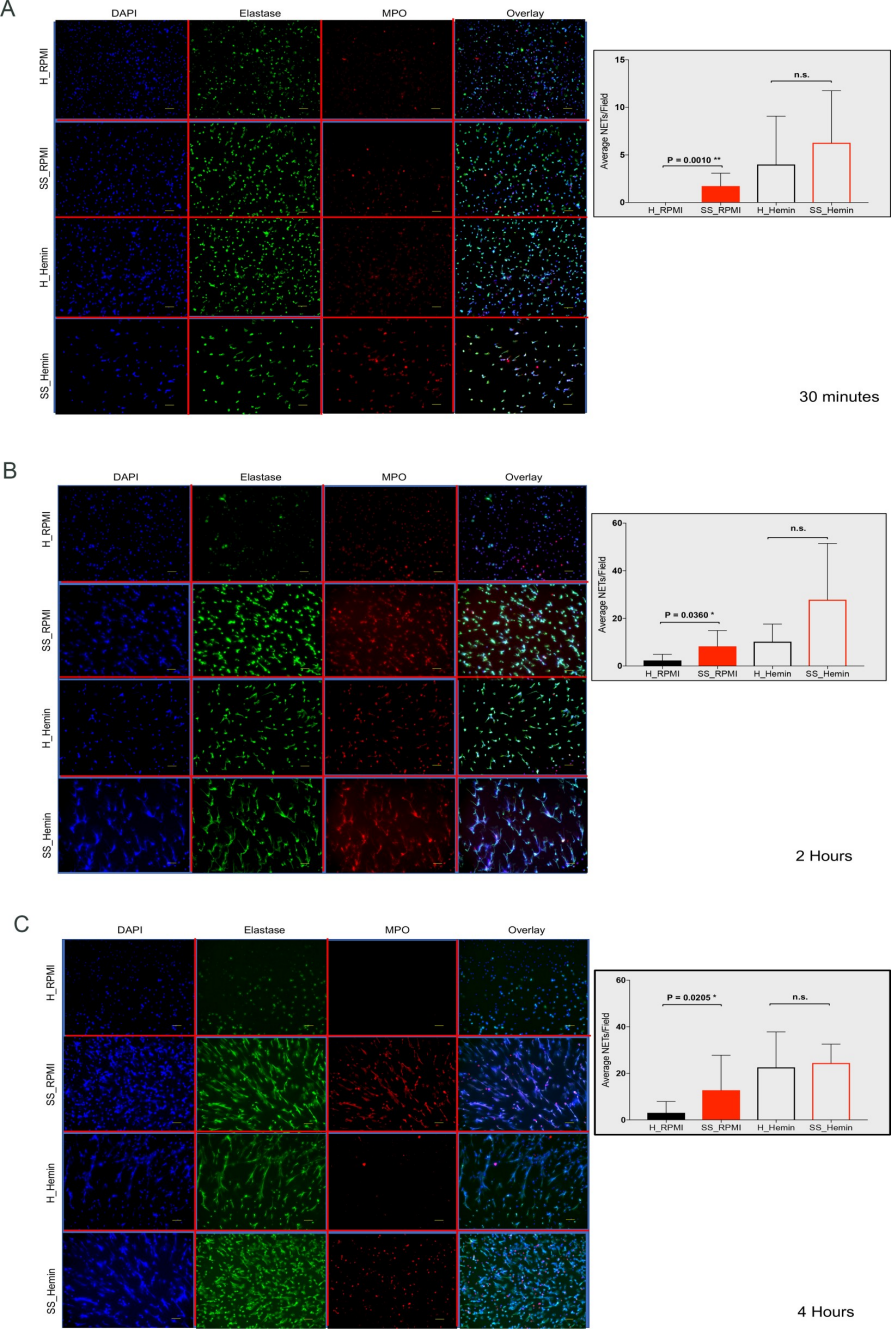
Ex vivo, untreated neutrophils from SCD patients are efficient at producing NETs in the absence of a specific trigger. Representative immunofluorescence microscopy data and NETs count at 30 minutes (A), 2 hours (B) and 4 hours (C). Scale bar is 50 μm. Purified and rested neutrophils from healthy donors (H) and from SCD patients at steady state (SS) were incubated with RPMI or 20 μM hemin. Neutrophils were fixed with 4% PFA and then stained for elastase, MPO and DNA. NETs production was counted in at least 10 acquired fields and shown as average NETs per field ± S.D. Significance was calculated with an unpaired Mann-Whitney test.

We confirmed the observations made using the imaging flow cytometry technology with immunofluorescence microscopy. Triple positive DNA-elastase-MPO strands (NETs) were counted in neutrophils from 9 healthy donors and 8 patients with SCD, and presented as average number of NETs per field ± standard deviation. At 30 minutes SCD neutrophils showed a wide-spread NETotic phenotype (enlarged nuclei co-localized with elastase) and even produced NETs (1.7±5), while these responses were not observed in the healthy neutrophils. Addition of hemin induced comparable responses between the SCD and healthy neutrophils (Fig 2A).

Within 2 hours in the absence of any trigger, there was a significant increase in the number of NETs formed by the SCD compared to healthy neutrophils (8.27±6.63 vs 2.33±2.59, P = 0.0360*). Although, with hemin the neutrophils from the SCD donors produced more NETs than the healthy ones the increase was not statistically significant (27.83±23.65 vs 10.2±7.4) (Fig 2B). At 4 hours, neutrophils from the SCD patients continued to demonstrate greater efficiency in forming NETs without any treatment, 12.70±5.66 vs 2.00±4.88 for healthy neutrophils (P = 0.02*) (Fig 2C). At this time point hemin induced a robust and comparable NETs production in both the SCD (21.00±8.11) and the healthy neutrophils (22.5±15.2). Taken together, our results confirm the presence of an intrinsic NETosis-prone phenotype in SCD neutrophils that appeared not to be enhanced by the hemin addition (S2 Fig).

We also investigated how the neutrophils from the SCD patients would respond to other stimuli known to cause NETs production. The average number of NETs per field produced by the neutrophils from the SCD patients following LPS 1 μg/ml stimulation was 4.37±3.63 while healthy neutrophils produced 4.37±3.63. With a lower hemin concentration (5 μM), the average NETs production was 5.78±4.23 vs 7.2±8.04 from healthy neutrophils. Similar fatigued response was also observed following LPS stimulation. NETs production was also not increased after treatment with the lower hemin concentration (S5 Fig). The average number of NETs per field produced by the neutrophils from the SCD patients following LPS stimulation was 4.37±3.63 while healthy neutrophils produced 4.37±3.63. With hemin 5 μM average production was 5.78±4.23 vs 7.2±8.04 from healthy neutrophils. These data showed that neutrophils from SCD donors did show a blunted NETs response to multiple stimuli.

### Sickle cell disease neutrophils are primed for degranulation

**Fig 3:**
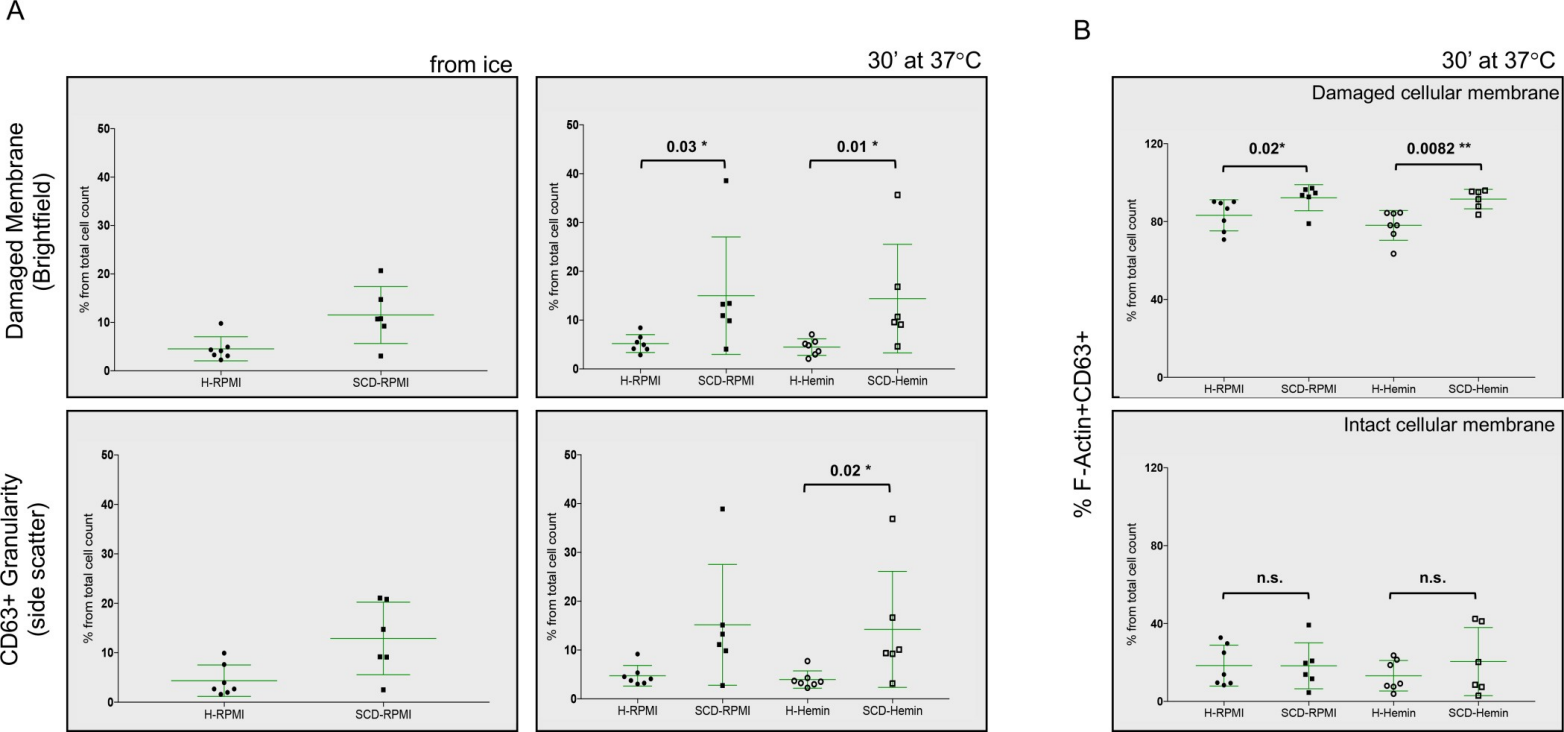
SCD neutrophils show increased degranulation-associated cellular changes, in the absence and presence of a stimulus. (A) In IFC, standard deviation versus modulation and SSC (side scatter) were used to asses cellular membrane damage and granularity in neutrophils from healthy controls (H) and that from SCD patients in steady-state (SCD) immediately following the purification step (left panels) or after 30 minutes incubation in RPMI or 20 μM hemin (right panels). (B) Purified and rested neutrophils from healthy controls (H) and that from SCD patients in steady-state (SCD) were left in RPMI or treated with 20 μM hemin for 30 minutes and percentage of the F-actin/CD63 double positive neutrophils with normal multi-lobulated nuclei that had damaged cell membranes (upper panel) or intact cell membranes (lower panel) was determined. (Healthy N = 7; SS N = 6). Data presented as dot plots±S.D., significance calculated with an unpaired Mann-Whitney test.

In the NETs assays we observed that a bigger fraction of the SCD neutrophils displayed a morphology consistent with damaged membrane compared to healthy neutrophils, both with IFC and microscopy (Fig 1 and S3A Fig). We suggest that this was due to ongoing degranulation, as cytoplasmic granules released their content by fusing with the cell outer membrane. While the mechanism(s) of neutrophil degranulation are not completely understood, the remodeling of the F-actin-scaffolded cytoskeleton and microtubules assembly appears to be involved in the recruitment of the granules to cell membrane. [18, 19] Tetraspanin CD63 is found only in the highly toxic neutrophil azurophilic granules containing amongst others elastase, MPO, defensins, cathepsins and can serve as a degranulation marker. To follow on intracellular changes related to degranulation we used IFC to track the cellular localization of F-actin and CD63 in resting and hemin-treated neutrophils from 7 healthy donors and 6 SCD patients. Prior to any treatment, cell membrane damage was already significantly increased in SCD neutrophils compared to their healthy counterparts (Fig 3A). The difference in the cell membrane texture between SCD and healthy neutrophils remained significant whether they were left untreated or hemin-treated for 30 minutes, P = 0.03* and P = 0.01*, respectively (Fig 3A).

**Fig 4:**
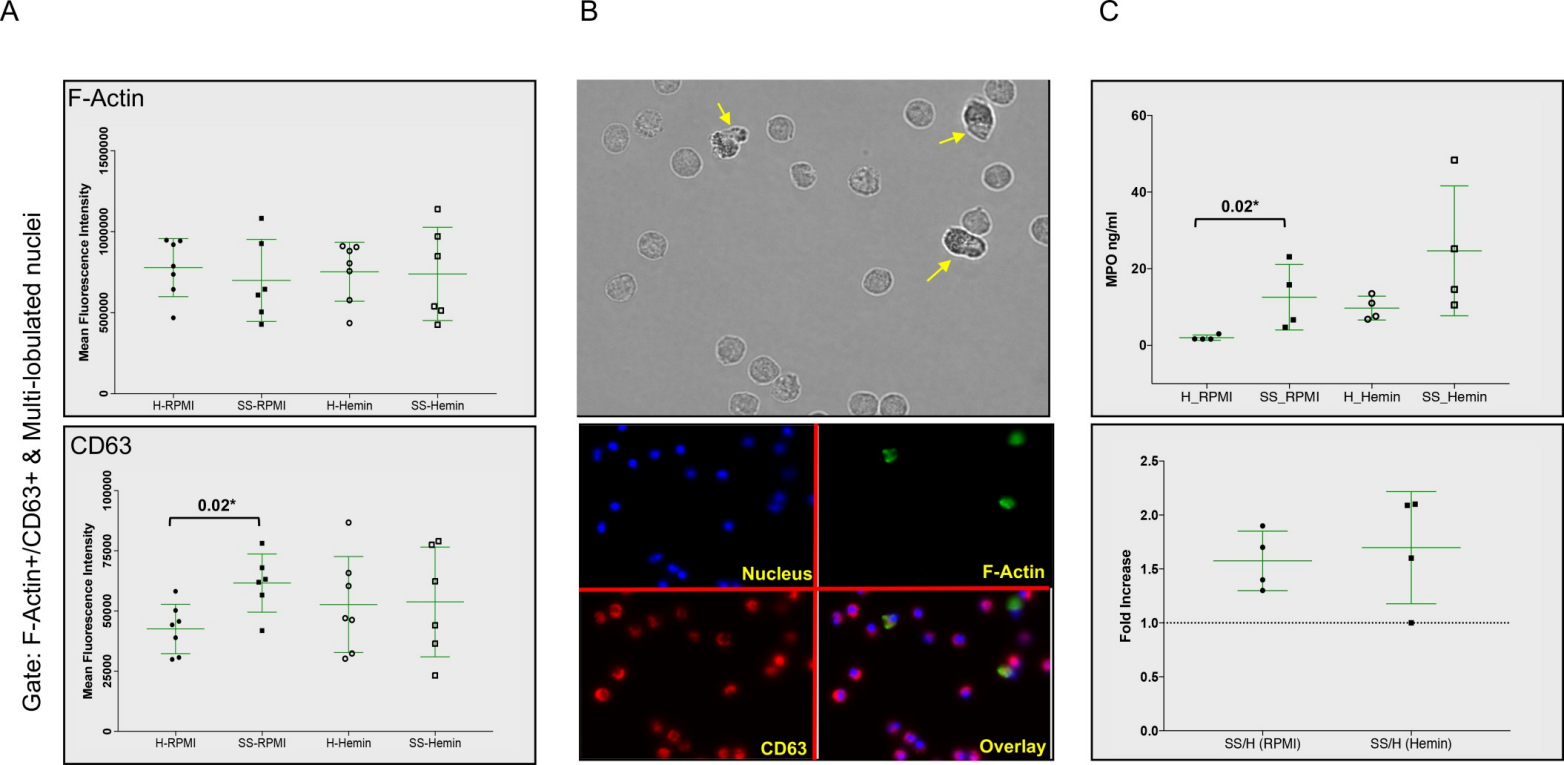
F-actin and CD63 co-localization associates with increased MPO release in SCD neutrophils. (A) Purified and rested neutrophils from healthy donors (H) and SCD patients in steady state (SS) were left untreated in RPMI as control or treated with 20 μM hemin. Median Fluorescence Intensity of F-Actin (upper panel) and CD63 (lower panel) in F-Actin/CD63 double positive neutrophils with normal multi-lobulated nuclei is shown. (Healthy N = 7; SCD N = 6). (B) Z-stack stills of SCD neutrophils left in RPMI for 30 minutes showing neutrophils with roughed cell membranes (yellow arrows, upper panel). Staining for DNA (blue), F-Actin (green) and CD63 (red) is presented (lower panel). (C) MPO release was assayed by ELISA following 30 minutes incubation with or without the hemin stimulus (H (Healthy) N = 4; SS (SCD) N = 4). Degranulation response was assayed in terms of MPO ng/ml plasma (upper panel), or fold increase over healthy neutrophils (lower panel). Data presented as dot plots±S.D., significance calculated with an unpaired Mann-Whitney test.

Addition of hemin appeared to enhance granularity in SCD neutrophils (Fig 3A). SCD samples contained more cells with damaged membranes that also had normal, multi-lobulated nuclei and were double positive for F-actin and CD63, after either RPMI or hemin incubation compared to healthy neutrophils (Fig 3B, P = 0.02* and P = 0.0082**, respectively). To determine how F-Actin and CD63 were recruited in these cells we looked at their median fluorescence intensity in the double positive F-Actin+/CD63+ gate. F-Actin signal intensity was comparable in healthy and SCD neutrophils, with or without hemin, but the CD63 fluorescence was higher in the untreated SCD neutrophils (Fig 4A, P = 0.0221*).

Neutrophils from SCD patients with rough plasma membranes had a depleted CD63 pool as compared to healthy ones, in the absence of a trigger or after hemin addition (S3B Fig, P = 0.03*). Taken together these data showed that neutrophils from SCD patients were more efficient in co-recruiting F-Actin and CD63, even without a stimulus, strongly suggesting that the SCD neutrophils could release their azurophilic granules spontaneously.

We further verified this hypothesis by measuring the MPO released from healthy and the neutrophils from the SCD donors after 30 minutes with and without hemin. Neutrophils from SCD patients were better at degranulating when left untreated (P = 0.02*) than when treated with hemin (Fig 4C).

## Discussion

NETs can contribute damage to the host in various ways [20–23] and one question of interest is whether neutrophils from patients with SCD are primed to produce NETs. Indirect assays that use plasma from SCD patients to induce response in healthy neutrophils cannot specifically address this issue. In a cross-sectional study using neutrophils isolated directly from patients with SCD in steady state and ethnic-matched healthy donors, we quantified the ability of the neutrophils to form NETs ex vivo, in absence and presence of an SCD-specific stimulus. Free circulating heme, common in highly hemolytic environments, has been associated directly (in mouse) and indirectly (in humans), with NETs formation in SCD. We asked whether a high concentration of the oxidized, stable form of heme (hemin 20 μM), was a potent NETs inducer.

Our results demonstrated that neutrophils from SCD patients receiving HU retained an increased functional behavior even at their basal, steady state. Whilst their count was reduced with HU therapy, these neutrophils still had an enhanced ability to form NETs and release their azurophilic granules ex vivo, in the absence of an inducer. Also, the kinetics of these cells’ response to hemin, was faster than that observed in the healthy neutrophils. We showed that the ex vivo SCD neutrophils underwent histone H4 citrullination fast (within 7 minutes), in the absence of a stimulus, suggesting that the SCD environment had already primed these cells for NETosis (Fig 1). Untreated SCD neutrophils already showed light NETs production compared to healthy neutrophils within 30 minutes and the increased production was maintained for all the 4 hours of observations (Fig 2). This type of intrinsic ability for NETs production has been previously reported in autoimmune conditions, such as rheumatoid arthritis (RA) and systemic lupus erythematosus (SLE), where there is continual recurring inflammatory activity.[24, 25] The presence of NETs has been associated with deleterious effects in various inflammatory pathologies, including sepsis,[26] transfusion-related acute lung injury,[27] vasculitis,[28] atherosclerosis,[29] and type I diabetes.[30] Because NETs can scaffold the formation of a clot or promote hypercoagulability by enhancing production of tissue factor and thrombin[31, 32] their prothrombotic properties are of particular interest in SCD.

Mechanistically, NETs formation requires production of reactive oxygen species (ROS) by mitochondria or NADPH oxidase, and translocation of azurophilic enzymes to nucleus and histone citrullination by PAD4.[33, 34] While we did not check ROS production in the context of our NETs experiments, it has been shown that the SCD environment is a strong promotor of NADPH oxidase-mediated superoxide production.[35] Because MPO translocation to the nucleus has been associated with NETosis [36], we used IFC to determine whether DNA and MPO co-localized in neutrophils with normal or decondensed nuclei, with or without hemin treatment (S4 Fig). We did find that hemin addition for 7 minutes caused a significant increase in DNA/MPO co-localization in neutrophils from the SCD patients compared with their healthy counterpart (P = 0.02*), indicating an ability for a faster response to the stimulus. Using microscopy, we also observed that hemin-untreated SCD neutrophils showed DNA-elastase co-localization prior to the formation of the DNA strands, while no such process was observed in their healthy counterparts (Fig 2A). Because previous data observed strong heme-mediated NETs response[16] the mild increase in the hemin-induced responses we observed in our study was unexpected at first. However, it did make sense in the context of the high spontaneous level of activation in the neutrophils from the SCD patients, likely responsible for the stimulus fatigue we observed. A similar fatigued response was also observed following LPS stimulation and NETs production was not increased after treatment with a lower hemin concentration (5 μM) (S5 Fig).

We identified other features of neutrophils activation that could be detrimental in the sickle inflammatory environment. Release of antimicrobial and proteolytic contents from primed neutrophils’ granules makes them both efficient pathogen fighters and a danger to the host. There are at least three different subsets of granules in neutrophils, that differ not only by the content, but also by their mobilization ability in response to stimulation. Azurophilic granules (containing MPO, elastase and other enzymes with toxic potential) are formed first in myelopoiesis and generally undergo limited stimulus-induced exocytosis.[37] Our study underlines the priming of SCD neutrophils for degranulation and also emphasizes the dangerous potential of this process. In earlier study no increase in CD63 expression was observed in SCD steady state neutrophils suggesting no increased activity around their azurophilic granules.[15] However, in our study SCD neutrophils mobilized their CD63 more efficiently to F-Actin and this feature might contribute to their increase ability to degranulate (Fig 4A).

The SCD patients in out cohort had neutrophil amounts that were comparable to those found in the healthy donors (S1 Table). They also suffered frequent painful crisis and other complications such as acute chest syndrome (ACS) and neutrophils are known to contribute to these acute clinical events. [38]

While in our study we determined that neutrophils appear to remain responsive in the SCD environment under HU treatment, we did not identify a direct activator. It is well established that SCD patients usually have high levels of circulating pro-inflammatory factors, in addition with chemokines and adhesion molecules. This would likely subject neutrophils to constant activating signals. Further studies that would look at the neutrophil responses and the plasma levels of pro-inflammatory factors in the same patient would further enrich the knowledge and provide a base for better therapy approaches.

In summary our results demonstrate that although neutrophil number may be reduced in patients with SCD receiving hydroxyurea treatment, they remain detrimentally active and retain several of their features with damaging potential that can contribute to sickle cell pathophysiology. It is thus likely that a multitargeted approach that included neutrophils would be more efficient for a better control of SCD severity.

## Supporting information

S1 Materials and Methods (DOCX)

S1 Fig Data analysis strategy with imaging flow cytometry. **(A)** Gating strategy for the imaging flow cytometry analysis for the NETs and the early activation experiments. **(B)** Nuclear and cellular features analyzed with the IDEAS software.
(TIF)

S2 Fig Summary of NETs production from healthy neutrophils (H) and neutrophils from SCD patients at steady state (SS). Neutrophils were incubated for 2 hours and 4 hours with RPMI or 20 μM hemin (microscopy). (Healthy, (H) N = 8; SCD (SS), N = 9). Data presented as dot plots ± S.D., significance calculated with a Mann-Whitney test.
(TIF)

S3 Fig Morphological differences between healthy neutrophils in neutrophils from SCD patients. **(A)** Brightfield microscopy images at 20x magnification showing healthy neutrophils (H) and neutrophils from a SCD patient at steady state (SS) with dissimilar cell surface morphology following 2 hours incubation with RPMI (no stimulus). **(B)** Percentage of F-Actin-/CD63+ neutrophils with normal multi-lobulated nuclei following 30 minutes incubation with RPMI or 20 μM hemin.
(TIF)

S4 Fig Co-localization of DNA and MPO in neutrophils from SCD patients is faster following hemin treatment. Purified neutrophils from healthy donors (H) or patients with (SS) were left untreated in RPMI or treated with 20 μM hemin for the specified times (7, 15 or 30 minutes). Stimulation was stopped with PFA and the fixed cells were stained for CD66b, H4cit3, MPO and DNA. Co-localization of the DNA and MPO signals were determined with the Similarity feature, an IDEAS analysis feature that calculates the degree to which the two staining images correlated within the nuclear area. Variable number of experimental repeats were conducted for each time point (7 minutes: N = 7: 15 minutes: N = 11; 30 minutes: N = 15). Data presented as dot plots±S.D., significance calculated with Mann-Whitney test.
(TIF)

S5 Fig Neutrophils from healthy donors or SCD patients show similar NETs response following 4 hours of treatment with hemin and LPS. Purified and rested neutrophils from 4 healthy donors (Healthy) and 4 SCD patients at steady state (SS) were treated as shown for 4 hours. Neutrophils were fixed with 4% PFA and then stained for elastase, MPO and DNA. NETs production was counted in at least 10 acquired fields and is shown as average NETs per field ± S.D. Significance was calculated with an unpaired Mann-Whitney test.
(TIF)

S1 Table Contribution of the neutrophils from healthy donors and SCD patients to experiments with details of white cell, neutrophil counts and clinical data. **(Table A)** Healthy donors list. NETosis IFC: Visualization of nuclear and cellular changes in fixed neutrophils sequentially stained for CD66B, H4cit3, MPO and DNA. NETosis microscopy: Visualization of DNA-scaffolded strands using BZ-X710 All-in-One Fluorescence Microscope after staining with elastase-Alexa Fluor 488, MPO-PE and DAPI. Early activation refers to 2 approaches: 1) visualization using IFC for degranulation-related assays in which fixed neutrophils were permeabilized and stained with anti-CD63-APC, ActinGreen488 and Hoechst and 2) MPO assays in which MPO content in supernatant from hemin-treated and un-treated neutrophils was assayed by ELISA. Several of the healthy volunteers (*) donated samples for studies on more than one occasion and their WBC and PMN is an average of the lab results on all dates. **(Table B)** SCD patients list. Experiments were conducted as described for the healthy donors. PHT–pulmonary hypertension; ACS–acute chest syndrome. Neutrophils from the patients in bold italic were used for the MPO degranulation tests. Subjects 3, 5, and 12 received blood transfusion between 4 and 8 weeks prior to blood sampling.
(XLSX)

S2 Table Nuclear and cellular features in neutrophils from healthy donors and steady state sickle cell patients. All stats for experimental data presented in Fig 1.
(TIF)
