# Supplementary material for: Neutrophils remain detrimentally active in hydroxyurea-treated patients with sickle cell disease
Source: PLoS One. 2019 Dec 23;14(12):e0226583. doi: 10.1371/journal.pone.0226583 (PMC6927657; doi:10.1371/journal.pone.0226583)
Supplement: S1 Materials and Methods — (DOCX) [file pone.0226583.s001.docx]

**Supporting information**

**Materials and methods.**

**Reagents, buffers and antibodies.**

Hemin was from Sigma (cat # H9039). Stock 32% Paraformaldehyde (PFA) Aqueous Solution, EM Grade was from Science Services (cat # E15714). Porcine skin gelatin was from Sigma (Type A, cat # G1890). BD Cytofix/Cytoperm (cat # 554722) and BD Perm/Wash 10x (cat # 554723) were from BD Biosciences.

Antibodies for Imaging Flow Cytometry (IFC): anti-human CD66B PE conjugated, clone G10F5 (Biolegend, cat # 305106) used at 5 μl per test; unconjugated primary antibody rabbit polyclonal anti-histone H4, citrulline 3 (H4cit3, EMD Millipore, cat # 07-596), used at 1:50 dilution. Secondary antibody goat anti-Rabbit IgG, DyLight 680 (Thermo Fisher, cat # 35568) used at 1:500 dilution. Hoechst 33342 (BD Pharmingen, cat #561908) diluted 1:1000.

Antibodies for immunofluorescence microscopy: unconjugated primary antibody rabbit polyclonal anti-neutrophil elastase (anti-NE, Abcam, cat # ab21595) used at 1:1000 dilution. Secondary antibodies goat anti-rabbit IgG Alexa Fluor-488 cat #A11008 or goat anti-rabbit IgG Alexa Fluor-594 cat #A11037 (Thermo Fisher Scientific, Life Technologies) for NE detection used at 1:500 dilution. Anti-human MPO, PE conjugated (clone REA491, Miltenyi Biotec, cat # 130-107-178) used at 1.5μg/ml. All working dilutions for above antibodies were prepared in sterile 2% BSA. Wash buffer for IFC and microscopy: 2% BSA in 1xDPBS, no calcium, no magnesium, + 2mM EDTA, filtered through 0.44 μm filters.

Blocking buffer for IFC and microscopy: sterile 3% BSA in 1xDPBS, no calcium, no magnesium, + 0.2% porcine skin gelatin.

Permeabilization buffer for microscopy: sterile 2% BSA in 1xDPBS, no calcium, no magnesium, + 0.005% Tween.

**NETs staining for microscopy.**

Purified neutrophils from healthy controls and patients with sickle cell anemia were plated in poly-L-Lysine coated glass chambers and allowed to rest for 30 minutes prior to the stimulation step. Treatment was stopped with PFA (final concentration 4%) for 20 minutes at room temperature (RT). Chambers were washed at least 4 times with PBS between the staining steps. Permeabilization buffer for microscopy was added for 15 minutes at RT. Primary rabbit polyclonal anti-neutrophil elastase antibody was added over night. Staining with the secondary antibody Alexa Fluor 594 and MPO-PE were conducted together at RT for 1 hour the next day. Finally, chambers were removed and slides were mounted in ProLong Gold Antifade Reagent with DAPI (Therrmo Fisher) for DNA staining and allowed to dry for 48 hours at 4°C before NETs visualization and quantification using a BZ-X710 All-in-One Fluorescence Microscope (Keyence).
