# Supplementary figures and images for: Neutrophils remain detrimentally active in hydroxyurea-treated patients with sickle cell disease
Source: PLoS One. 2019 Dec 23;14(12):e0226583. doi: 10.1371/journal.pone.0226583 (PMC6927657; doi:10.1371/journal.pone.0226583)

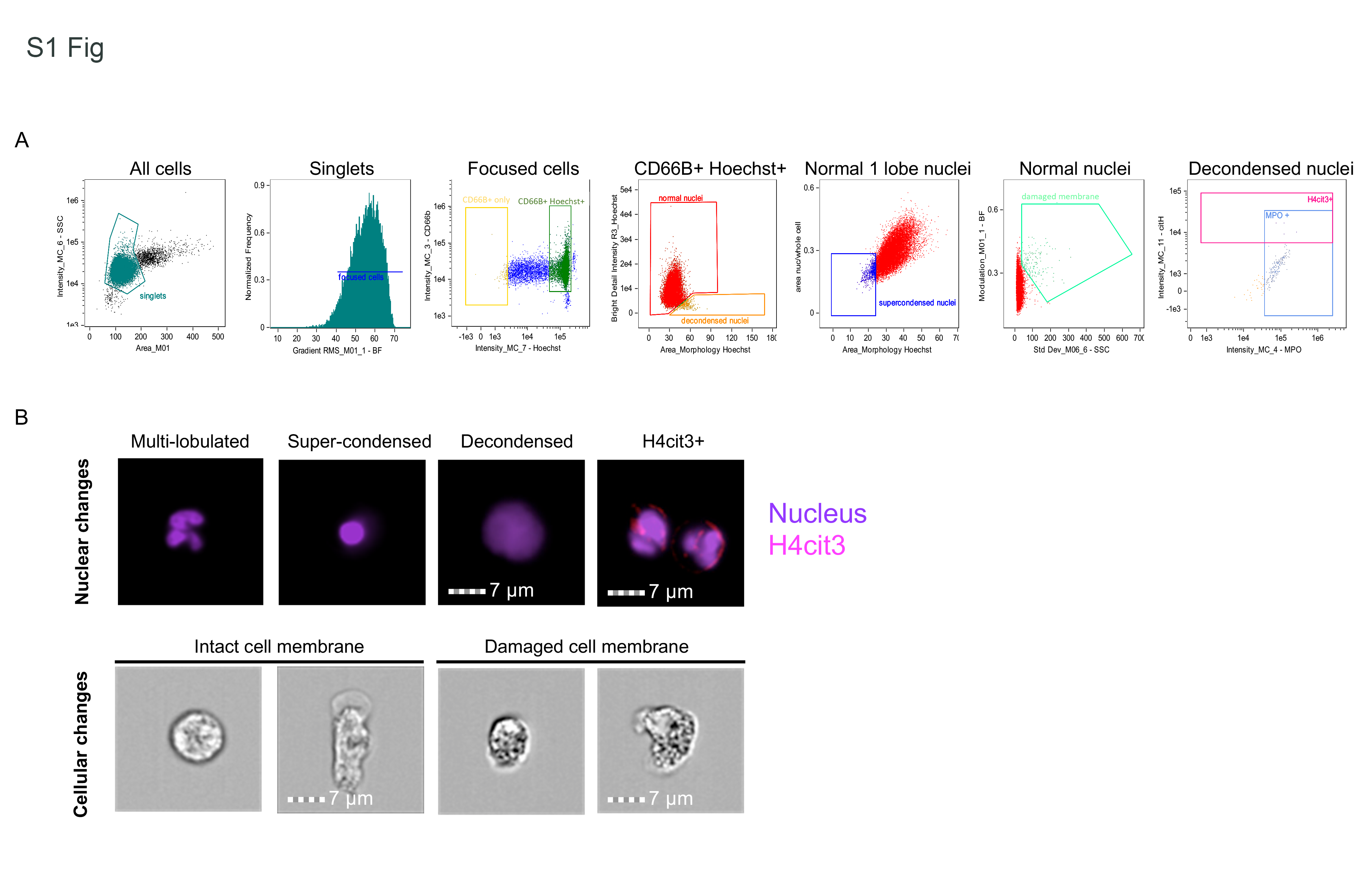

Supplement: S1 Fig — (A) Gating strategy for the imaging flow cytometry analysis for the NETs and the early activation experiments. (B) Nuclear and cellular features analyzed with the IDEAS software. (TIF) [file pone.0226583.s002.tif]

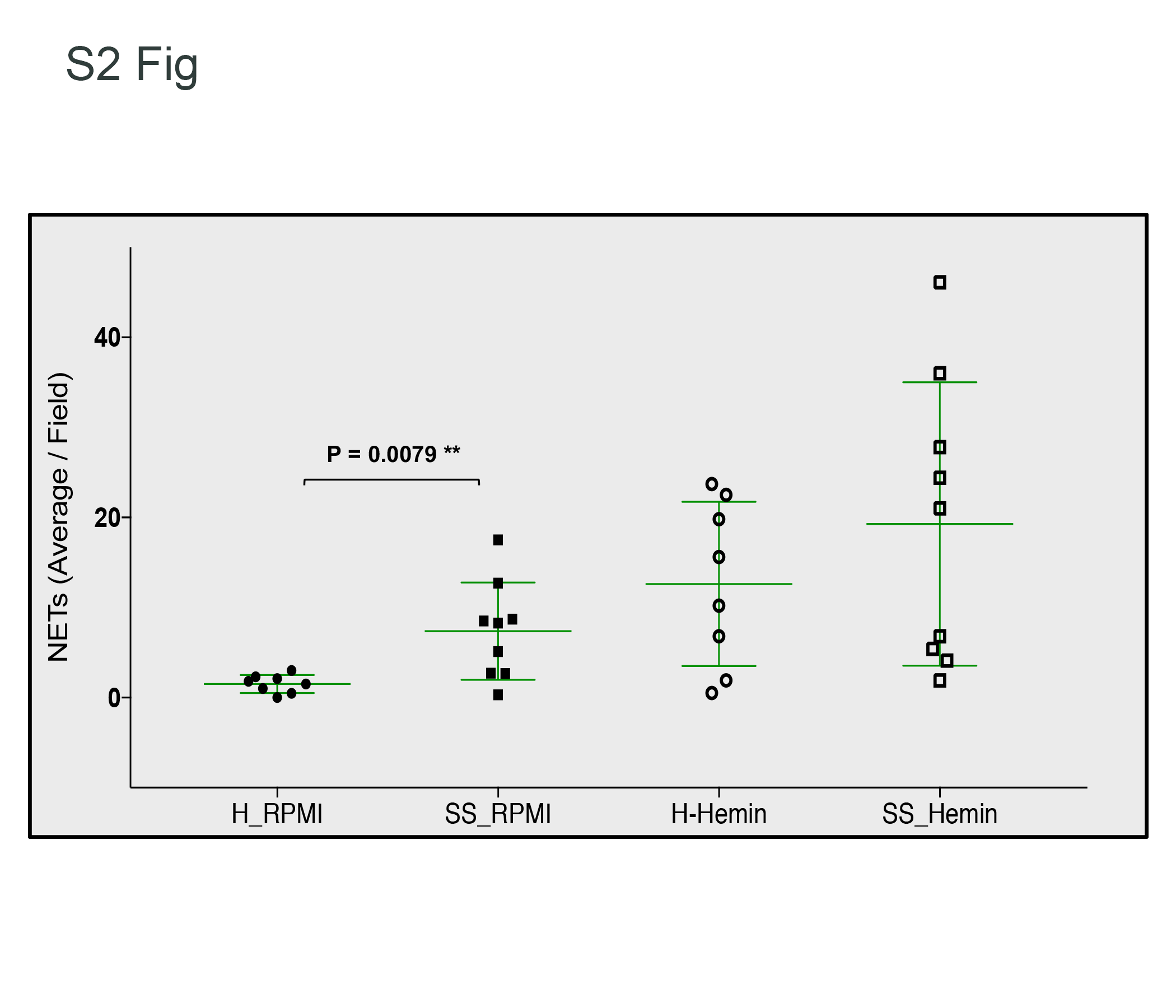

Supplement: S2 Fig — Neutrophils were incubated for 2 hours and 4 hours with RPMI or 20 μM hemin (microscopy). (Healthy, (H) N = 8; SCD (SS), N = 9). Data presented as dot plots ± S.D., significance calculated with a Mann-Whitney test. (TIF) [file pone.0226583.s003.tif]

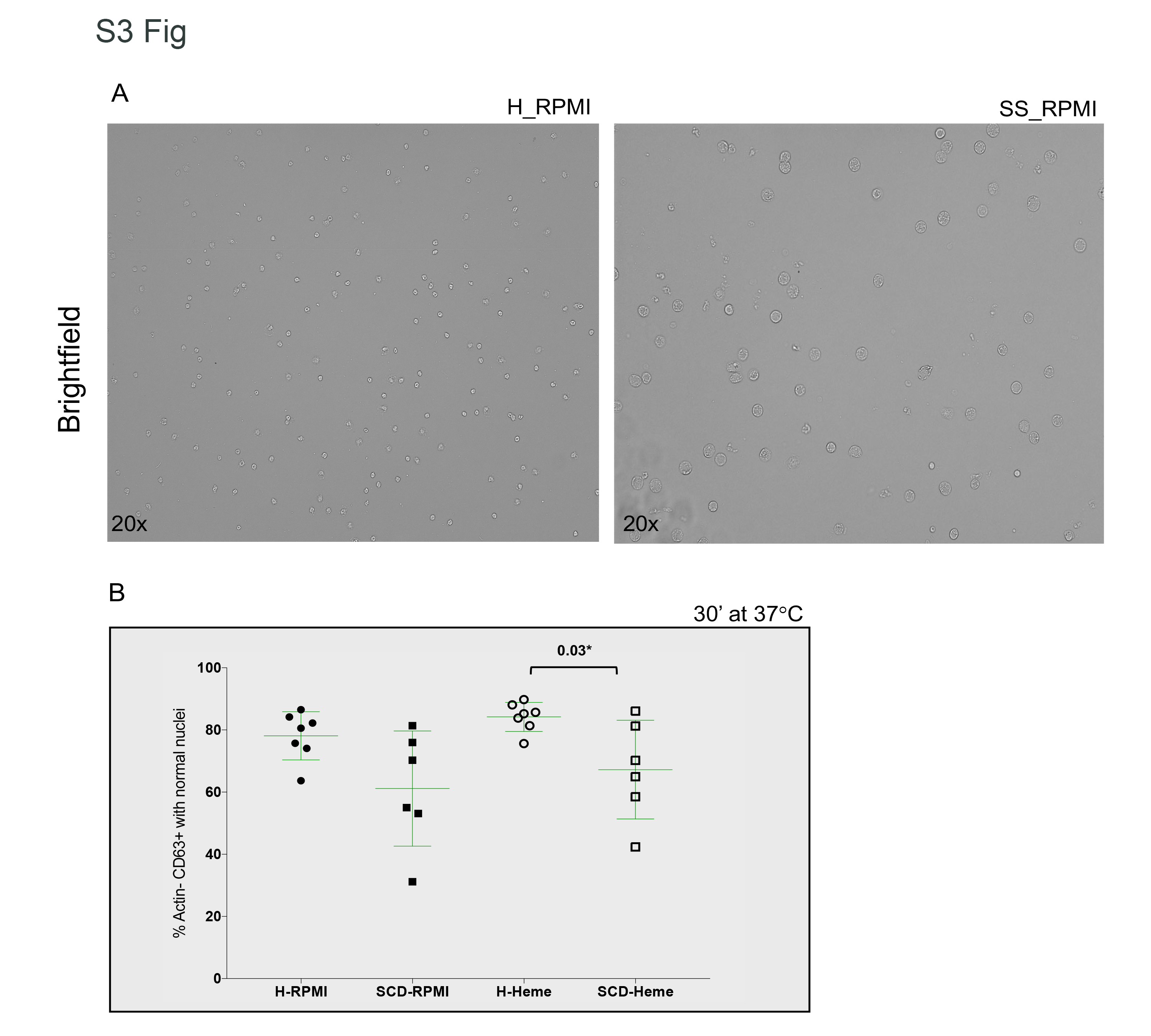

Supplement: S3 Fig — (A) Brightfield microscopy images at 20x magnification showing healthy neutrophils (H) and neutrophils from a SCD patient at steady state (SS) with dissimilar cell surface morphology following 2 hours incubation with RPMI (no stimulus). (B) Percentage of F-Actin-/CD63+ neutrophils with normal multi-lobulated nuclei following 30 minutes incubation with RPMI or 20 μM hemin. (TIF) [file pone.0226583.s004.tif]

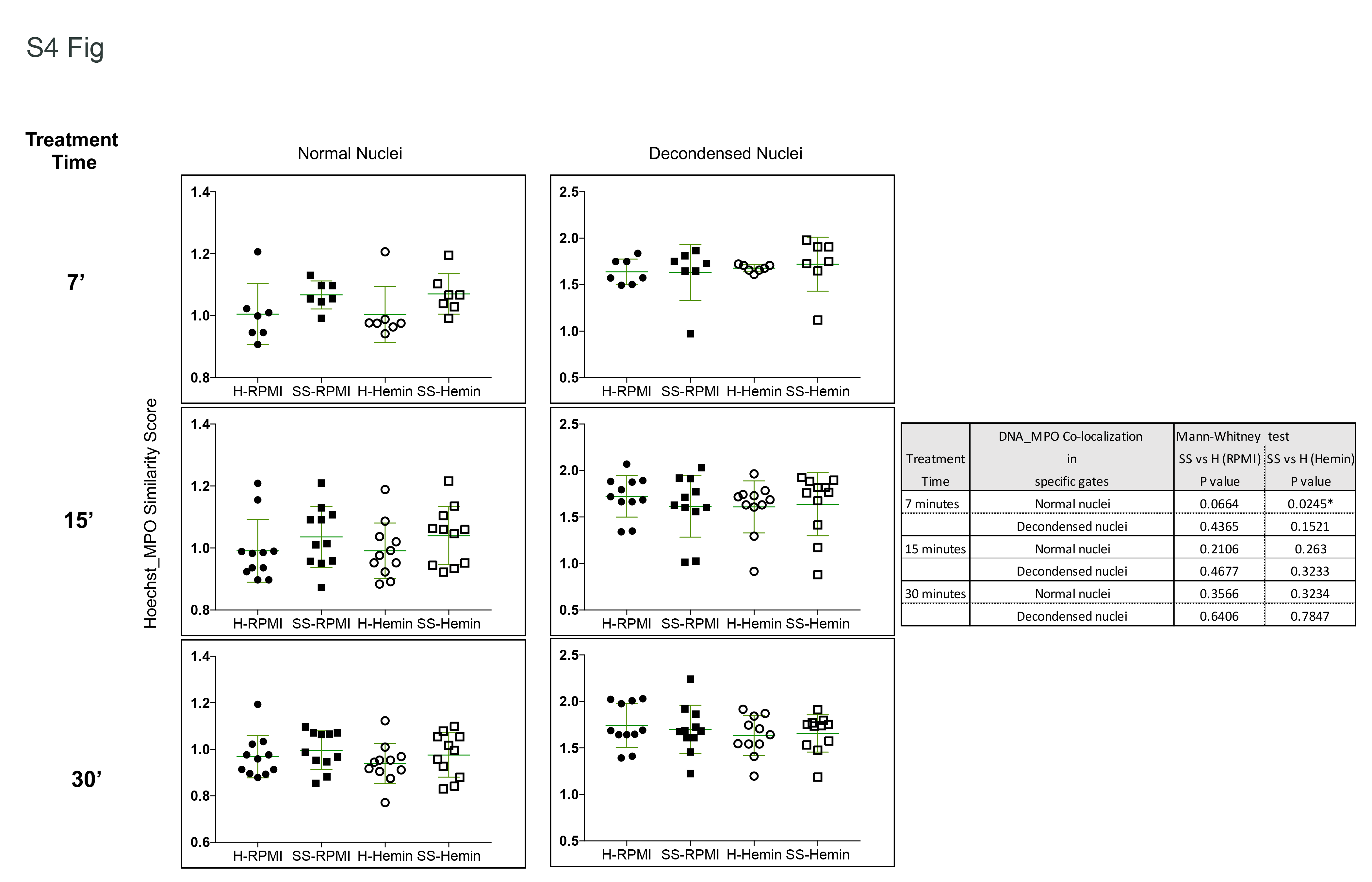

Supplement: S4 Fig — Purified neutrophils from healthy donors (H) or patients with (SS) were left untreated in RPMI or treated with 20 μM hemin for the specified times (7, 15 or 30 minutes). Stimulation was stopped with PFA and the fixed cells were stained for CD66b, H4cit3, MPO and DNA. Co-localization of the DNA and MPO signals were determined with the Similarity feature, an IDEAS analysis feature that calculates the degree to which the two staining images correlated within the nuclear area. Variable number of experimental repeats were conducted for each time point (7 minutes: N = 7: 15 minutes: N = 11; 30 minutes: N = 15). Data presented as dot plots±S.D., significance calculated with Mann-Whitney test. (TIF) [file pone.0226583.s005.tif]

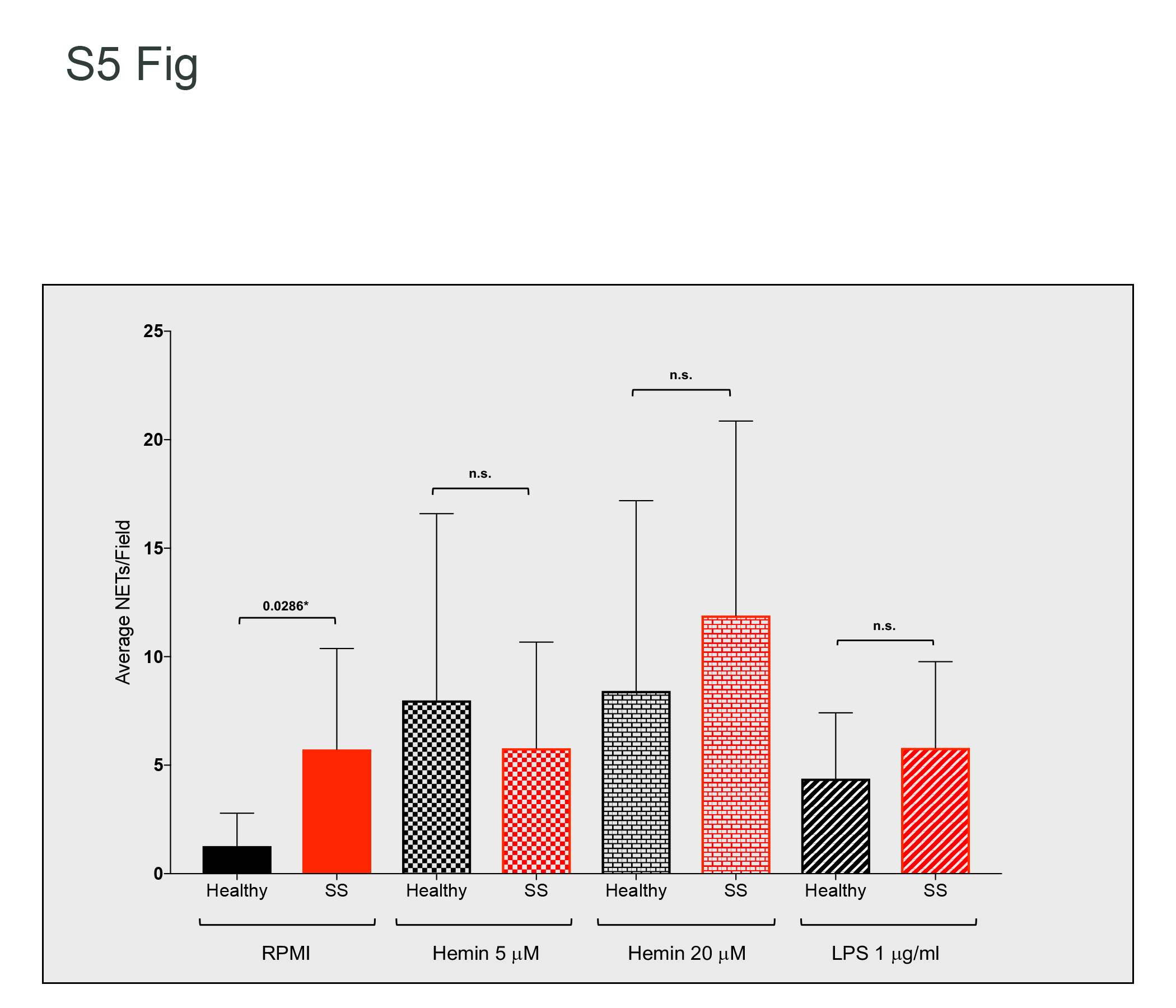

Supplement: S5 Fig — Purified and rested neutrophils from 4 healthy donors (Healthy) and 4 SCD patients at steady state (SS) were treated as shown for 4 hours. Neutrophils were fixed with 4% PFA and then stained for elastase, MPO and DNA. NETs production was counted in at least 10 acquired fields and is shown as average NETs per field ± S.D. Significance was calculated with an unpaired Mann-Whitney test. (TIF) [file pone.0226583.s006.tif]

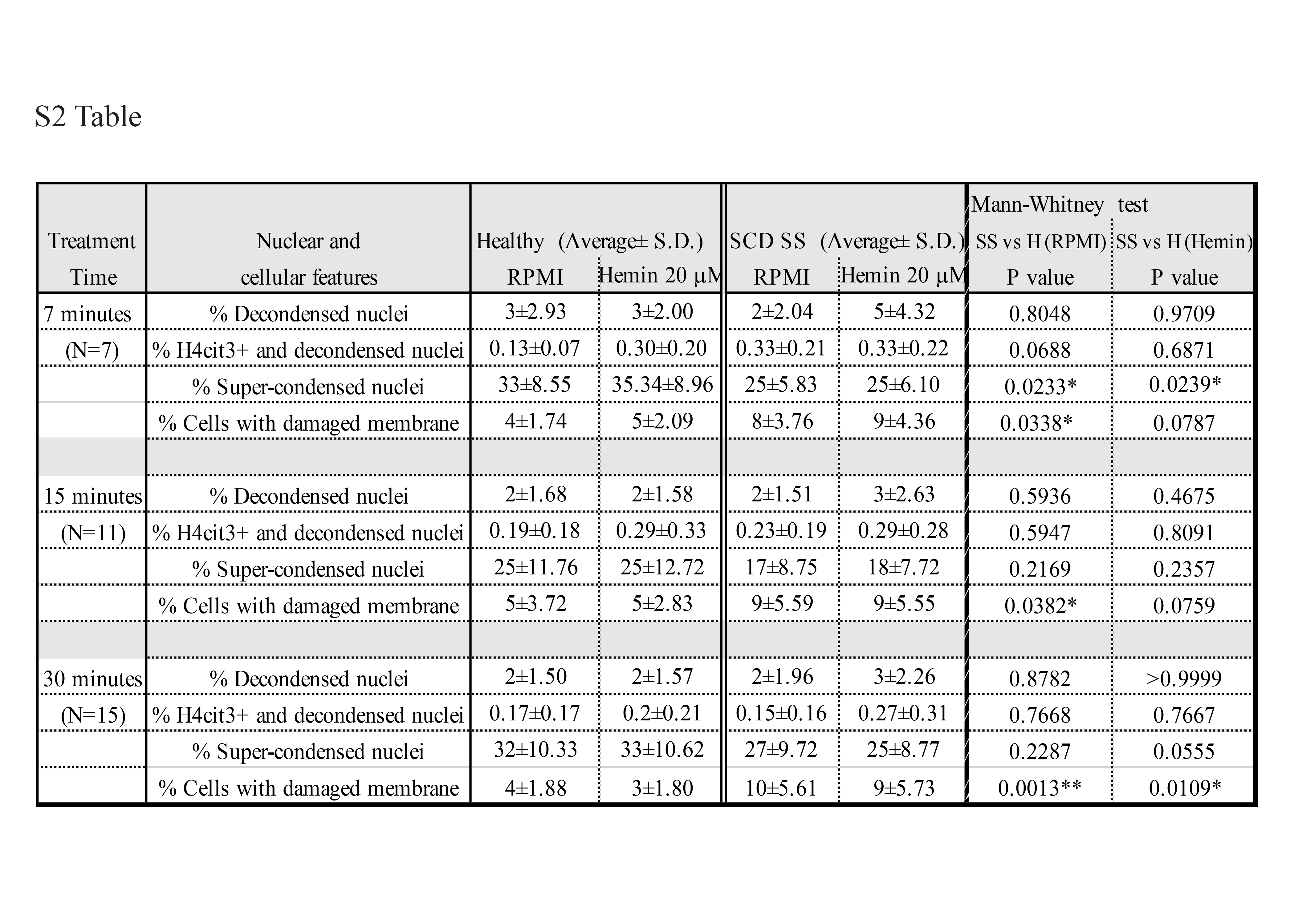

Supplement: S2 Table — All stats for experimental data presented in Fig 1. (TIF) [file pone.0226583.s008.tif]
